# How Important Are Rats As Vectors of Leptospirosis in the Mekong Delta of Vietnam?

**DOI:** 10.1089/vbz.2014.1613

**Published:** 2015-01-01

**Authors:** Hoang Kim Loan, Nguyen Van Cuong, Ratree Takhampunya, Bach Tuan Kiet, James Campbell, Lac Ngoc Them, Juliet E. Bryant, Bousaraporn Tippayachai, Nguyen Van Hoang, Serge Morand, Vo Be Hien, Juan J. Carrique-Mas

**Affiliations:** ^1^ Institute Pasteur, Ho Chi Minh City, Vietnam.; ^2^ Oxford University Clinical Research Unit, Ho Chi Minh City, Vietnam.; ^3^ Nuffield Department of Medicine, Oxford University, Oxford, United Kingdom.; ^4^ Sub-Department of Animal Health, Dong Thap, Vietnam.; ^5^ Armed Forces Research Institute of Medical Sciences, Bangkok, Thailand.; ^6^ ISEM (CNRS, IRD, UM2), Université Montpellier 2, France.

**Keywords:** Leptospirosis, Mekong Delta, Vietnam, Rats

## Abstract

Leptospirosis is a zoonosis known to be endemic in the Mekong Delta of Vietnam, even though clinical reports are uncommon. We investigated leptospira infection in rats purchased in food markets during the rainy season (October) (*n*=150), as well as those trapped during the dry season (February–March) (*n*=125) in the region using RT-PCR for the *lipL32* gene, confirmed by 16S rRNA, as well as by the microscopic agglutination test (MAT). Results were compared with the serovar distribution of human cases referred from Ho Chi Minh City hospitals (2004–2012) confirmed by MAT (*n*=45). The MAT seroprevalence among rats was 18.3%. The highest MAT seroprevalence corresponded, in decreasing order, to: *Rattus norvegicus* (33.0%), *Bandicota indica* (26.5%), *Rattus tanezumi* (24.6%), *Rattus exulans* (14.3%), and *Rattus argentiventer* (7.1%). The most prevalent serovars were, in descending order: Javanica (4.6% rats), Lousiana (4.2%), Copenageni (4.2%), Cynopterie (3.7%), Pomona (2.9%), and Icterohaemorrhagiae (2.5%). A total of 16 rats (5.8%) tested positive by RT-PCR. Overall, larger rats tended to have a higher prevalence of detection. There was considerable agreement between MAT and PCR (kappa=0.28 [0.07–0.49]), although significantly more rats were positive by MAT (McNemar 29.9 (*p*<0.001). MAT prevalence was higher among rats during the rainy season compared with rats in the dry season. There are no current available data on leptospira serovars in humans in the Mekong Delta, although existing studies suggest limited overlapping between human and rat serovars. Further studies should take into account a wider range of potential reservoirs (*i.e*., dogs, pigs) as well as perform geographically linked co-sampling of humans and animals to establish the main sources of leptospirosis in the region.

## Introduction

Leptospirosis is a zoonotic infection of global relevance, caused by pathogenic spirochetes within the genus *Leptospira*. Humans become infected through cuts and skin abrasions or by drinking contaminated water. Symptoms range from mild, influenza-like illness to severe infection with renal and hepatic failure, pulmonary distress, and death, although most infections are thought to be asymptomatic (Adler and de la Pena Moctezuma 2010). Leptospirosis is thought to be endemic in Vietnam and other countries in the region (Laos, Cambodia, and Thailand), with seasonal peaks of incidence during the rainy season and outbreaks related to flooding events (Victoriano et al. 2009). Leptospira organisms are notoriously difficult to detect and to culture, therefore the most commonly used diagnostic methods are based on detecting a serological response. The microscopic agglutination test (MAT), based on detecting agglutinating antibodies against the infecting serovar using a dark-field miscrocope, is considered the gold standard for serological tests. Panels of live leptospires belonging to locally circulating serovars are maintained in the laboratory and used as antigens for the MAT (World Health Organization 2003).

Leptospirosis was first identified in Vietnam in 1930 (Vaucel 1936). Early studies (1967–1968) on sera from foreign troops indicated that leptospirosis was then the most common cause (20%) of acute fever of unknown origin in Vietnam (Berman et al. 1973). Studies of acute jaundice in southern Vietnam from 1993 to 1997 (*n*=550) identified leptospira infection among 7% patients in An Giang (Mekong Delta) and 4% patients in Ho Chi Minh City (HCMC). Eleven patients were identified as being leptospira-infected by either MAT or PCR tests. MAT (titer ≥1:400) identified three patients positive with serovar (sv.) Bataviae. Serological reactions also identified sv. Hurstbridge (two patients), Pyrogenes (one), and Hardjo bovis (one), although titers were lower (≤1:100) (Laras et al. 2002). Sero-studies (1995) among healthy human populations in Tien Giang province (Mekong Delta) identified sv. Bataviae (21.7%), Panama (15.2%), Icterohaemorrhagiae (13.7%), and Australis (8.7%) as the most common (Van et al. 1998).

Rodents are considered a major reservoir of human leptospira infection (Sarkar et al. 2002, Athanazio et al. 2008, Meerburg et al. 2009, Desvars et al. 2012). Recent studies on rats trapped in Hanoi and Hai Phong port (northern Vietnam) indicated a 22% prevalence of anti-leptospira antibodies by enzyme-linked immunosorbent assay (ELISA) using lipL32 protein as antigen; all seropositive rats were *Rattus norvegicus*, and none of the six *R. tanezumi* rats investigated tested positive (Koma et al. 2013).

Little is known about the prevalence of infection and current levels of circulation of leptospira among rats in the Mekong Delta of Vietnam, which has markedly different climate and agro-ecological characteristics compared with northern Vietnam. We carried out a study in the Mekong Delta with the aim of characterizing leptospira infection (by real-time PCR [RT-PCR]) and seroprevalence (by MAT) in rats at two different time points (dry and rainy seasons). These results were evaluated in the context of the epidemiological features of confirmed human infections referred by HCMC hospitals.

## Materials and Methods

### Geographic and climatic features

The Mekong Delta, covering about 40,000 square kilometres, is a low-level plain situated at <3 meters above sea level and criss-crossed by a maze of canals and rivers. The area is farmed intensively with pockets of natural vegetation. The area has a tropical monsoon climate, with temperatures ranging from 25°C to 28°C and slight annual variations. The rainy season is from May to October. Rice is the predominant crop, and rats are commonly trapped after each harvest and sold for food in markets.

### Rat collection and processing

Rats were purchased from markets in five (Dong Thap, Tien Giang, An Giang, Vinh Long, and Can Tho) of 12 Mekong Delta provinces (October, 2012). Rat trapping was conducted over 10 consecutive days (between February 26 and March 8, 2013) on a range of locations in Dong Thap province, including farm sites raising poultry and pigs (*n*=20 locations), the edge of rice fields and fruit groves (*n*=6), and the Gao Giong Natural Park (*n*=4), a tropical forest with large numbers of canals and native cajuput trees. Trapping and identification procedures are described in the manuscript by Loan et al. (submitted). Rats were euthanized by an overdose of inhalant anesthetic (isoflurane) following American Veterinary Medical Association (AVMA) guidelines (Anonymous 2013). Immediately after been culled, blood (1–5 mL) was obtained from rats by heart puncture. Immediately after collection, serum was separated from the clot using a centrifuge, and 100 μL of serum was used for MAT. One kidney was extracted from each rat, and special precautions were taken to avoid cross-contamination. The kidneys were immediately frozen at −80°C.

### Human surveillance data from southern Vietnam

Serum samples from patients referred to HCMC hospitals with suspected leptospirosis are routinely sent to Pasteur Institute in HCMC (PI-HCMC) where they are investigated by the MAT (see below). Data from 2004–2012 on sample submissions to PI-HCMC of suspect leptospirosis cases, with dates of onset, age, and sex data, as well as number of confirmed infections were available for analysis.

### Real-time PCR and *Leptospira* species identification

Rats were investigated for the presence of leptospiral DNA in their kidneys. DNA was extracted from kidney tissue using a Wizard Genomic DNA Purification Kit (Promega, Fitchburg, WI). A RT-PCR assay targeting the *lipL32* gene of pathogenic leptospires (McAvin et al. 2012) was performed. RT-PCR targeting the *Leptospira* spp. 16S rRNA gene (Smythe et al. 2002) was used to confirm positive samples. PCR amplification and PCR-directed sequencing of the whole 16S rRNA gene was performed using a previously published protocol (Matthias et al. 2005). Full-length 16S rRNA gene sequences of *Leptospira* spp.–positive rats were analyzed with reference sequences retrieved from the GenBank database (www.ncbi.nlm.nih.gov/genbank/). Strains were classified as pathogenic, nonpathogenic, and intermediate on the basis of DNA sequence identity to previously classified *Leptospira* spp. genotypes (Morey et al. 2006). Multiple sequence alignment and phylogenetic tree construction were performed using MEGA 5.1 program (Tamura et al. 2011).

### Microscopic agglutination test

The presence of antibodies against *Leptospira* spp. was investigated in rat and human sera using the MAT with a panel of 23 pathogenic serovars thought to be endemic in Vietnam. In addition, *L. biflexa* sv. Patoc (a saprophytic serovar) was used to indicate nonspecific reactions to serovars not included in the panel, as recommended by World Health Organization (WHO) guidelines (2013) (see Table S1; Supplementary Data are available at www.liebertonline/vbz). The panel used represented 16 major serogroups most frequently found in Southeast Asia (supported by the WHO Collaborating Centre for Leptospirosis, Paris). Agglutination was examined using a dark-field microscope. The MAT technique was performed according to WHO guidelines. A titer was defined as the highest dilution giving 50% agglutination in comparison with that of the negative control. Rat serum was considered positive when a titer of 1:20 or higher was obtained with at least one strain (Vanasco et al. 2001). A case of human leptospirosis was defined as an individual with clinical symptoms consistent with lepospira infection and a MAT titer ≥1:400.

### Statistical considerations and data analyses

The surveys were carried out as part of a wider study investigating a wide range of pathogens in rats. The study aimed at investigating ∼300 rats. This sample size theoretically allows the estimation of a 7% MAT prevalence (based on previous results on rats in Vietnam) within 20% of the true prevalence and 80% certainty. Results of the MAT and RT-PCR tests were compared using the kappa statistic and the McNemar chi-squared test. The distribution of specific serovars in rats and between humans and rats were compared using the Pearson correlation test. Pairwise correlations between seropositivity for different serovars in rats were plotted using a correlogram. Weight data from *R. norvegicus* rats were used to estimate age (in weeks) from published growth curves for male and female *R. norvegicus* rats (Eisen 1976). The data used corresponded to progeny of breeding rats fed on a restricted diet.

The hazard (probability of infection per unit time) was calculated by fitting a (left censored) null survival model to the MAT seroprevalence data by age (Dohoo et al. 2003). Incidence rate (*IR*) was extrapolated from the seroprevalence data (or cumulative incidence) (*CI*) (*CI*=1−exp(−*IR*)). The duration of infection (*D*) was estimated from incidence rate (*IR*) and prevalence (*P*) of infection (from RT-PCR test results) (*D*=*P*/*IR*) (Dohoo et al. 2003). All calculations were carried out assuming 100% sensitivity and specificity of diagnostic tests. To investigate the association between rat size and prevalence of infection (as determined by RT-PCR), rat body and head length for each species were divided into four quantiles and the proportion positive estimated for each quantile. The association between leptospira MAT seropositivity and the rat species, body length, and type of rat (purchased vs. trapped) was investigated by fitting a multivariable logistic regression model. The type of rat was considered to be a proxy of seasonality, because rats were purchased at the end of the rainy season (October) and trapped during the dry season (February–March). Multivariable model building was carried out in a stepwise forward fashion starting with variables with the lowest *p* value in univariable analyses (Hosmer and Lemeshow 2004). The interaction between all main effects remaining in the model was investigated. All analyses were carried out using the R statistics program (www.r-project.org) with packages ‘epicalc’, ‘survival’, and ‘corrgram’.

## Results

### *Leptospira* prevalence by RT-PCR (rats)

All 275 rats (150 purchased rats and 125 trapped rats) were investigated by RT-PCR for leptospira. A total of 16 rats tested positive: 5.8% (95% confidence interval [CI] 3.1–8.6%) (Table 1). *Bandicota indica* and *R. norvegicus* rats had the highest prevalence of infection (10.8% and 6.9%, respectively) (Table 2). The probability of a RT-PCR positive result was 0/70 (0%), 3/70 (4.3%), 5/64 (7.8%), and 8/71 (13.1%) for rats belonging to the first to the fourth size quantile of head and body length, respectively, suggesting increasing prevalence associated with size (χ^2^ for trend=8.94; *p*=0.03). There were no differences in prevalence by sex (*p*=0.62).

**Table 1.** Overall Results of MAT and RT-PCR Testing of 275 Rats (Mekong Delta, Vietnam) (October, 2012–March, 2013)

	*Market survey (2012)*	*Field survey (2013)*	*All rats (2012–2013)*
MAT	26/120 (21.7%)	18/121 (14.8%)	44/241 (18.2%)
RT-PCR	12/150 (8.0%)	4/125 (3.2%)	16/275 (5.8%)

MAT, microscopic agglutination test; RT-PCR, real-time PCR.

**Table 2.** RT-PCR Test Results of 275 Rats Investigated for Leptospira (Mekong Delta, Vietnam, 2012–2013)

	*All rats*		*Purchased rats*		*Trapped rats*	
	*No. pos./ No. tested*	*%*	*No. pos.*	*%*	*No. pos.*	*%*
*B. indica*	7/65	10.8	7/44	15.9	0/21	0.0
*R. norvegicus*	2/29	6.9	0/9	0.0	2/20	10.0
*R. argentiventer*	5/104	4.8	5/88	5.7	0/16	0.0
*R. exulans*	0/16	0.0	—	—	0/16	0.0
*R. tanezumi*	2/61	3.3	0/9	0.0	2/52	3.8
All	16/275	5.8	12/150	8.0	4/125	3.2

RT-PCR, real-time PCR; Pos., positive.

### Phylogenetic analyses of *Leptospira* spp. DNA sequences (rats)

**FIG. 1.**
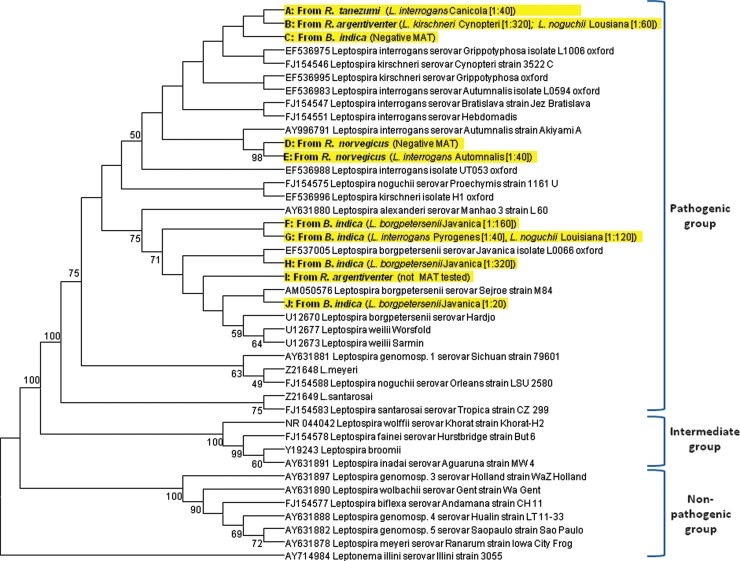
Phylogenetic tree showing relationship between sequences of 10 *Leptospira* isolates analyzed for the amplified region of 16S rRNA. The rat species from which leptospiral DNA was detected as well as the microscopic agglutination test (MAT) titers in those rats are indicated.

A total of 10 DNA sequences (of 16) yielded a clean and strong signal in the DNA chromatogram of sequencing results. All 10 *Leptospira* spp. DNA sequences from rats fell within the pathogenic group. Results from the analysis for the 10 sequences obtained (*A* through *J*) as well as MAT-positive rats are presented in Figure 1. Sequence analysis revealed five isolates with high homology to *L. interrogans* (FJ154551 sv. Hebdomadis) (99.5–99.8% identity). The remaining isolates were closely associated with *L. borgpetersenii* (EF537005 sv. Javanica) with percent identity ranging from 99.8% to 99.9%. DNA sequences of 16S rRNA genes from *D* and *E* were identical. The16S rRNA gene sequence of isolates in the *L. borgpetersenii* group were almost identical to each other, with only a single nucleotide mutation (T→A).

### MAT survey results (rats)

Thirty-four rats had insufficient serum, and therefore 241/275 rats were MAT tested. A total of 44 rats (18.2% [95% CI 13.4–23.1%]) tested positive by MAT (Table 1). Although the crude seropositivity was higher for purchased rats compared with trapped rats, this difference was not statistically significant (χ^2^=1.43, *p*=0.231). The highest MAT seroprevalence corresponded, in decreasing order, to: *R. norvegicus* (33% [95% CI 15.6–51.1%]), *B. indica* (26.5% [14.2–38.9%]); *R. tanezumi* (24.6% [13.4–23.2%]), *R. exulans* (14.3% [0–32.6%]) and *R. argentiventer* (7.1% [1.6–12.7%]). There were no differences in prevalence by sex (*p*=0.30). Given the low numbers of rats in each type of trapping site (farms, rice fields, forest land) strata it was not possible to demonstrate any difference in leptospira prevalence between sites.

**FIG. 2.**
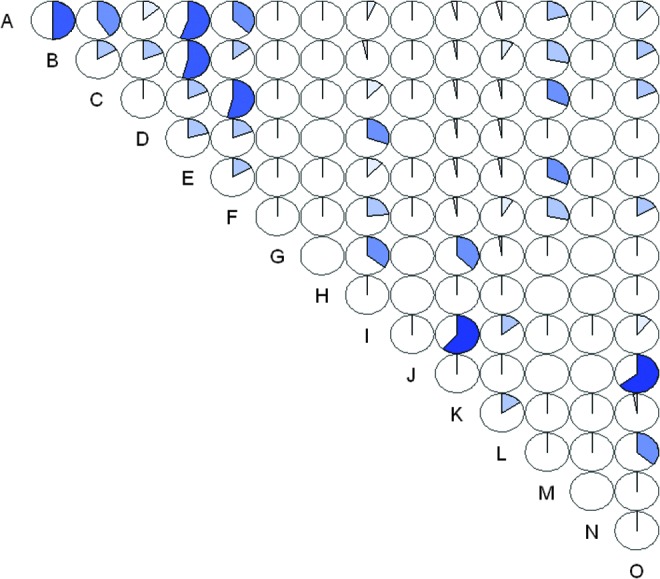
Pairwise correlation between reactions to all leptospira serovars tested in 242 rats tested by the microscopic agglutination test (MAT) (Mekong Delta, Vietnam) (2012–2013). The magnitude of the pie represents the strength of the Pearson correlation coefficient. Key. A=Copenhageni; B=Icterohaemorrhagiae; C=Pomona; D=Australis; E=Pyrogenes; F=Canicola; G=Automnalis; H=Bataviae; I=Lousiana; J=Panama; K=Cynopterie; L=Javanica; M=Hurstbridge; N=Tarasovi; O=Patoc.

Reactions to a total of 15 serovars belonging to six *Leptospira* spp. were identified (Table 3). Three of the four rats with sequences close to *L. interrogans* had MAT titers suggesting previous exposure to *L. borgpetersenii* sv. Javanica. The proportion of positive rats reacting against one, two, three, and four or more serovars was 52.3%, 20.5%, 18.2%, and 9.1%, respectively. The most common reactions identified were: sv. Javanica (12 rats), Copenhageni (10), Lousiana (10), Cynopteri (nine), Australis (six), Canicola (six), Icterohaemorrhagiae (six), Pomona (five), Pyrogenes (five), and Automnalis (four). The strongest pairwise correlation in prevalence was observed between sv. Panama and Patoc (Pearson's correlation [cor.] 0.64), sv. Lousiana and Cynopterie (cor. 0.62), sv. Copenhageni and Pyrogenes (cor. 0.55), sv. Pomona and Canicola (cor. 0.54), sv. Icterohaemorrhagiae and Pyrogenes (cor. 0.50), and sv. Copenhageni and Icterohaemorrhagiae (cor. 0.50) (all *p* values <0.05). There was little evidence of cross-reactivity between serovars belonging to *L. noguchii* (sv. Lousiana and Panama) (cor. 0.010; *p*=0.83) or *L. borgpetersenii* (sv. Javanica and Tarassovi) (cor. 0.014; *p*=0.820). Reactivity against the saprophytic *L. biflexa* sv. Patoc had moderate to high (0.36–0.64) correlation with sv. Panama and Javanica, and small (0.16–0.18) but significant (*p*<0.05) correlation with sv. Icterohaemorrhagiae, Pomona and Canicola (*p*<0.05) (Fig. 2). The MAT prevalence was considerably higher for rats in the fourth quartile of body size (19/52) (32.2%) compared with rats in the first to third quantile (14.4–16.1%) (χ^2^=6.98, *p*=0.008). There was a fair agreement between MAT and RT-PCR tests (kappa=0.29; [95% CI=0.19–0.39]; *Z*=5.63; *p*<0.001), although the MAT test detected significantly more positive rats compared with the RT-PCR (McNemar χ^2^=24.3; *p*<0.001).

**Table 3.** MAT Serological Responses to Leptospira Serovars (Reciprocal of Titer) among 241 Rats Investigated in the Mekong Delta, Vietnam (2012–2013)

			*Rat species*					
*Species*	*Serogroup*	*Serovar*	R. norvegicus (n*=27)*	B. indica (n*=49)*	R. tanezumi (n*=57)*	R. exulans (n*=14)*	R. argentiventer (n*=84)*	*All rats (*n*=241)*
*L. interrogans*	Icterohaemorrhagiae	Copenhageni	3 (40, 20, 20)	2 (40, 40)	5 (80, 80, 80, 40, 40)	1 (40)		10
	Icterohaemorrhagiae	Icterohaemorrhagiae	1 (160)		4 (320, 320, 320, 160)		1 (40)	6
	Pomona	Pomona	2 (80, 40)		2 (40, 40)		1 (40)	5
	Australis	Australis	1 (80)		4 (160, 40, 40, 40)	1 (80)		6
	Pyrogenes	Pyrogenes		1 (40)	3 (160, 40, 40)	1 (80)		5
	Canicola	Canicola	3 (40, 20, 20)	1 (20)	2 (80, 40)			6
	Automnalis	Automnalis	1 (40)	2 (80; 160)	1 (40)			4
	Bataviae	Bataviae	1 (40)		1 (40)			2
*L. noguchii*	Lousiana	Louisiana	2 (160, 20)	4 (320, 320, 320, 20)	2 (160, 160)		2 (640, 80)	10
	Panama	Panama			1 (40)			1
*L. kirschneri*	Cynopteri	Cynopteri		4 (320, 160,80,80)	2 (160,40)		3 (640, 320, 20)	9
*L. fainei*	Hurstbridge	Hurstbridge			2 (40, 40)			2
*L. borgpetersenii*	Javanica	Javanica	3 (640, 20, 20)	5 (320, 160, 40, 20, 20)	4 (640, 640, 320, 160)			12
	Tarassovi	Tarassovi	1 (20)	1 (20)				2
*L. biflexa*	Semaranga	Patoc	2 (40, 40)	1 (20)	2 (40, 40)			5
		Any serovar	9 (33.3%)	13 (26.5%)	14 (24.6%)	2 (14.3%)	6 (7.1%)	44 (18.3%)

MAT, microscopic agglutination test.

### Surveillance data (humans)

**FIG. 3.**
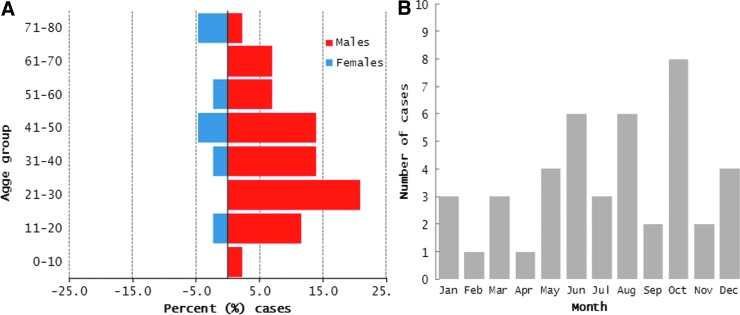
(**A**) Age and sex distribution among human cases of leptospirosis in southern Vietnam (2004–2012) (cases submitted to the Pasteur Institute of Ho Chi Minh City), confirmed using microscopic agglutination test (MAT) (titer 1:400 or higher). (**B**) Distribution of cases by month of diagnosis.

A total of 983 human samples were tested by the MAT between 2004 and 2012 (annual median=105 [interquartile range, IQR=81–138]). Of those, 45 (4.6%) were confirmed as leptospirosis, as they had clinical signs compatible with leptospirosis and were accompanied by a MAT titer of 1:400 or higher. Of 41 cases with gender data, 34 (82.9%) were males. Among male cases, 61.8% were aged between 21 and 50 years. A total of 30 (66.7%) cases were diagnosed during the rainy season (May to October in southern Vietnam) (Fig. 3). Eight of 45 cases (17.8%) had a reaction with a titer of 1:400 or higher against more than one serovar. The most common reactions were against sv. Bataviae (14 patients), Hurstbridge (14), Canicola (nine), Tonkini (nine), Cynopterie (five), Javanica (four), Cynopterie (five), Gryppotyphosa (five), Louisiana (four), and Icterohaemorrhagiae (four). In addition, reactions against sv. Australis, Hardjo, Copenhageni, and Pyrogenes were reported (two or less patients each). Unfortunately data on MAT reactions with titers of <1:400 were not available for analysis. There was no significant correlation between human surveillance serovar distribution and serovar distribution among the tested Mekong Delta rats (correlation=0.18; *p*=0.46).

### Risk factors for MAT seropositivity (rats)

*R. exulans* and *R. argentiver* rats were combined because they had the lowest overall MAT prevalence, and there was no significant difference in prevalence between both. Factors independently associated with an increased risk of testing positive were: (1) Rat species: *B. indica* (odds ratio [OR]=3.6), *R. norvegicus* (OR=6.0), and *R. tanezumi* (OR=3.9) (vs. *R. exulans* and *R. argentiventer* combined); (2) rat size (*i.e*., rats in the fourth quantile of body size) (OR=3.74); and (3) purchased rat (vs. trapped rat) (OR=4.6). No interaction factors were significant (Table 4).

**Table 4.** Multilevel Logistic Regression Model Showing Significant Factors for MAT Leptospira Prevalence in Rats (Mekong Delta, Vietnam, 2012–2013)

	*Univariable*			*Multivariable*^a^		
*Variable and level*	*OR*	*95% CI*	p *value*	*OR*	*95% CI*	p *value*
Rat species (baseline=*R. exulans* and *R. argentiventer*)	1.0	—	—	1.0	—	—
*B. indica*	3.2	1.2–8.2	0.017	3.6	1.4–9.6	0.007
*R. norvegicus*	5.6	1.9–16.5	0.002	6.0	2.0–18.2	0.002
*R. tanezumi*	3.7	1.4–9.4	0.007	3.9	1.5–10.3	0.005
Large rat (4^th^ quantile)	2.9	1.4–5.8	0.030	3.7	1.5–6.6	0.002
Purchased rat (baseline=trapped rat)	1.6	0.81–3.07	0.174	4.6	1.8–11.7	0.001

^a^ Model intercept=−4.062 (standard error [SE]=0.609).

MAT, microscopic agglutination test; OR, odds ratio; CI, confidence interval.

### Dynamics of infection (*R. norvegicus* rats)

**FIG. 4.**
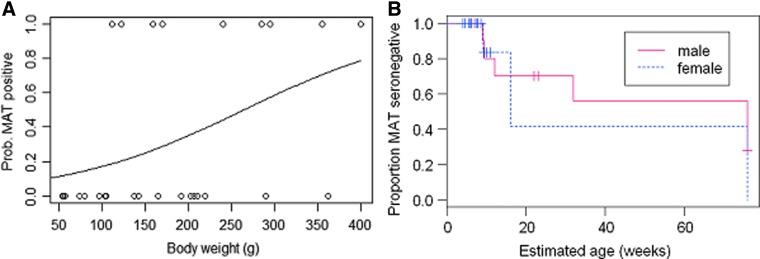
(**A**) Relationship between head and body weight (in grams) and probability of testing positive by the microscopic agglutination test (MAT) test. (**B**) Kaplan–Meier survival curves for male and female *R. norvegicus* rats (outcome=MAT seropositivity) (Survival indicates probability of being seronegative).

Data on seropositivity and the Kaplan–Meier survival curve for male and female *R. norvegicus* rats is presented in Figure 4. The estimated hazard rate was 0.0088 (per week) (95% CI=0.0067–0.0109) (equivalent to a probability of 0.46 per year; 95% CI=0.35–0.57). The estimated duration of infection (calculated from observed prevalence and incidence) was therefore 6.61 (95% CI=5.34–8.68) weeks.

## Discussion

Among Mekong Delta rats surveyed, sv. Javanica, Copenhageni, Lousiana, and Cynopteri were the most common serovars. In contrast, surveillance data from HCMC hospitals indicate that sv. Bataviae, Hurstbridge, and Canicola were most commonly associated with human leptospirosis during 2004–2012. These three serovars, although detected in Mekong Delta rats, were not predominant. However, *ad hoc* studies on rats carried out by PI-HCMC across southern Vietnam (mostly in provinces not including the Mekong Delta) over the period 2005–2012 indicated that sv. Bataviae and Hurstbridge were the two most common serovars detected in rats by MAT (data not shown). It is very likely that a large fraction of human cases referred to HCMC hospitals are from regions not represented in the areas included in the rat survey. This emphasizes the need to improve human surveillance for leptospirosis in Vietnam, for example, by collecting data on geographical origin of patients.

Furthermore, other humans, as well as domestic species, such as pigs and dogs, common in the Mekong Delta may also play a role in transmission of infection, and their role should be investigated. Seroprevalence studies on pigs in 10 provinces in the Mekong Delta indicated that sv. Bratislava, Icterohaemorrhagiae, Automnalis, Grippotyphosa, and Pomona were most common (Boqvist et al. 2002a, b). However, it is not entirely appropriate to compare these results with human and rat data, given that the MAT serovar panel in those studies was limited (*i.e*., did not include sv. Hurstbridge, Tonkini, and Cynopteri). The absence of rodent control programs on pig farms was a risk factor for pig herds becoming infected with sv. Javanica, but not other serovars such as Australis, Automnalis, Bratislava, or Icterohaemorrhagiae (Boqvist et al. 2002a), suggesting that rats may play an active role in dissemination of leptospirosis between pig farms. Because leptospirosis is a disease that causes important economic losses to the pig industry, these data should provide an impetus for enhanced rodent control programs on farms. The authors are not aware about any studies on leptospira in dogs in the Mekong Delta. Data from northern Thailand indicated 11% MAT seroprevalence in dogs, with the highest corresponding to sv. Bataviae, followed by Canicola (Meeyam et al. 2006), both of which are common as causes of humans leptospirosis in southern Vietnam.

Our results indicate moderate levels of leptospira infection among rats in the Mekong Delta as determined by RT-PCR (5.8%). These results are lower than the prevalence levels reported from southeast China (20–40% depending on RT-PCR method used) (Yalin et al. 2011), Thailand (15.3% using direct fluorescence) (Doungchawee et al. 2005), the Philippines (43% by culture and pulsed-field gel electrophoresis) (Villanueva et al. 2010), and Cambodia (RT-PCR) (11.6%) (Ivanova et al. 2012), but similar to levels reported in Malaysia (RT-PCR) (6.7%) (Benacer et al. 2013). However, differences in sample size, species distribution, as well as in laboratory methods for determining prevalence complicate comparisons across studies. For example, differences in performance of RT-PCR tests for *lipL32* and *G1/G2* genes have been reported, and these differences may also be serovar dependent (Yalin et al. 2011). Seroprevalence levels by MAT among Mekong Delta rats (∼18%), was similar to those in a recent study on rats from northern Vietnam (22%) using enzyme-linked immunosorbent assay (ELISA) (Koma et al. 2013), but higher than those reported for different locations in southern Vietnam other than the Mekong Delta over the 2005–2012 period (∼7% using MAT) (data not shown).

The demographic features of human cases from this study were comparable to other countries in Southeast Asia (Laras et al. 2002), with a clear predominance of cases among adult males. It has been suggested that in Vietnam and in Asia in general males are more commonly involved in fieldwork and may have more intimate contact with animals. However, this needs to be confirmed. The seasonal presentation may be a result of a combination of increased exposure in the rainy season with higher number of rodents that may have also increased prevalence of infection (Perez et al. 2011). The relatively low number of samples from suspect cases referred by doctors in southern Vietnam highlights the limited awareness among the medical community of this disease, which is thought to be highly endemic in the Mekong Delta (19% MAT human seropositivity in Tien Giang province) (Van et al. 1998).

We observed higher levels of detection using MAT compared with RT-PCR and a discrepancy between MAT titers and sequencing results. This suggests that leptospira infections in rats are not life long, and MAT titers are likely to indicate a previous (cleared) infection in most cases. In contrast, other studies have reported higher levels of detection of the pathogen, compared with seroprevalence, and the authors suggested that single infections may not be sufficient to trigger a serological response (Collares-Pereira et al. 2000, Pereira and Andrade 1988).

There were considerable differences in the levels of detection of leptospira in different rat species, with *R. norvegicus* and *B. indica* being the two most commonly infected species. Similar results have been reported in Thailand (Doungchawee et al. 2005) and Cambodia (Ivanova et al. 2012). It has been suggested that the observed differences in prevalence may be a reflection of differences in population densities, rather than intrinsic differences in susceptibility among rat species (de Faria et al. 2008).

We observed a higher prevalence of detection by RT-PCR among older rats, in agreement with studies in Asia (Ivanova et al. 2012, Wang and He 2013), suggesting long-term carriage and the absence of pathogenic effects of leptospira infection on rats (Athanazio et al. 2008). The estimated duration of leptospira infection among *R. norvegicus* rats (∼7 weeks) was considerably shorter than the duration of infection in rats experimentally inoculated using a high dose (10,000 *L. interrogans* serovar Copenhageni organisms) (>4 months) (Athanazio et al. 2008). However, in a previous report, rats infected with sv. Icterohaemorrhagiae maintained a renal carrier state for 220 days compared to 40 days for rats infected with sv. Grippotyphosa, suggesting considerable differences between serovars (Thiermann 1981). Therefore, it is possible that detection results may have biased the observed results toward longer-lasting serovars in rats.

The observed higher seroprevalence among rats purchased in markets compared with trapped rats is likely to be a reflection of the different time periods of the surveys. Whereas market rats were investigated at the end of the rainy season (October), rat trapping was conducted during the dry season (Feburary–March). Studies in Cambodia have also shown clear seasonality patterns, with higher prevalence corresponding to the flooding season (Ivanova et al. 2012).

In summary, we used RT-PCR and MAT to determine the prevalence of current and previous leptospira infection in rats in the Mekong Delta of Vietnam. We also noted some key features such as a higher prevalence among *R. norvegicus* and *B. indica* rats and an association between age and prevalence. Human surveillance data specific to the Mekong Delta are not available, and data for southern Vietnam indicate little overlapping with Mekong Delta rat serovars. However, given the great diversity of leptospira in different host species and the potential variations over time and space, more detailed studies involving co-sampling of humans as well as rats and a wider range of reservoirs in the same areas are required. These results should be used to encourage further surveillance of circulating serovars and episodes of clinical infection, and this knowledge should be used to define the appropriate tools for control.

## Supplementary Material

Supplemental data
