# Supplementary material for: How Important Are Rats As Vectors of Leptospirosis in the Mekong Delta of Vietnam?
Source: Vector Borne Zoonotic Dis. 2015 Jan 1;15(1):56–64. doi: 10.1089/vbz.2014.1613 (PMC4307199; doi:10.1089/vbz.2014.1613)
Supplement: Supplemental data [file Supp_Table1.pdf]

Supplementary Data

TABLE S1. PANEL OF ANTIGENS SET USED IN THE MICROAGGLUTINATION TEST

No	*Specie*	*Serogroup*	*Serovar*	*Strain*
1	*L. interrogans*	Australis	Australis	Ballico
2	*L. interrogans*	Autumnalis	Autumnalis	Akiyami A
3	*L. interrogans*	Bataviae	Bataviae	Van Tienen
4	*L. interrogans*	Canicola	Canicola	Hond Utrecht IV
5	*L. borgpetersenii*	Ballum	Castellonis	Castellon 3
6	*L. interrogans*	Icterohaemorrhagiae	Copenhageni	Wijnberg
7	*L. interrogans*	Pyrogenes	Pyrogenes	Salinem
8	*L. interrogans*	Icterohaemorrhagiae	Tonkini	LT 96 68
9	*L. interrogans*	Icterohaemorrhagiae	Icterohaemorrhagiae	Verdun
10	*L. kirschneri*	Cynopteri	Cynopteri	3522 C
11	*L. kirschneri*	Grippotyphosa	Grippotyphosa	Moskva V
12	*L. borgpetersenii*	Sejroe	Hardjobovis	Sponsela
13	*L. interrogans*	Hebdomadis	Hebdomadis	Hebdomadis
14	*L. borgpetersenii*	Javanica	Javanica	Veldrat Bataviae 46
15	*L. noguchii*	Panama	Panama	CZ 214 K
16	*L. biflexa*	Semaranga	Patoc	Patoc 1
17	*L. interrogans*	Pomona	Pomona	Pomona
18	*L. borgpetersenii*	Tarassovi	Tarassovi	Mitis Johnson
19	*L. weilii*	Tarassovi	Vughia	LT 09 68
20	*L. interrogans*	Sejroe	Hardjo	Hardjo Prajitno
21	*L. interrogans*	Sejroe	Saxkoebing	Mus24
22	*L. interrogans*	Canicola	Canicola	Chiffon
23	*L. noguchii*	Louisiana	Louisiana	LSU 1945
24	*L. fainei*	Hurstbridge	Hurstbridge	BUT6
